# A first public dataset from Brazilian twitter and news on COVID-19 in Portuguese

**DOI:** 10.1016/j.dib.2020.106179

**Published:** 2020-08-18

**Authors:** Tiago de Melo, Carlos M.S. Figueiredo

**Affiliations:** a LSI–Laboratório de Sistemas Inteligentes, Escola Superior de Tecnologia, Universidade do Estado do Amazonas, Brazil

**Keywords:** COVID-19, Pandemic, Dataset, Twitter, News, Portuguese

## Abstract

In this data article, we provide a collection of 3,925,366 tweets and 18,413 online news around the online discussion about COVID-19 in Brazil. The data from Twitter were collected through Twitterscraper Python library and we considered a set of keywords in Portuguese regarding to COVID-19. In order to facilitate the identification of tweets that have hashtags, media and retweets for researchers or data enthusiasts, we created three specific datasets for each of these categories. The news on COVID-19 was collected from the UOL portal, the most popular Brazilian website. All the data were gathered from January to May, 2020. These datasets can attract the attention from communities such as data science, social science, natural language processing, tourism, infodemiology, and public health.

**Specifications Table**

**Table:** 

Subject	Social Science, Health Informatics, Computer Science
Specific subject area	Covid-19 related online and social media mining for understanding the main discussed topics and effects on people's life.
Type of data	Text (CSV-formatted)
How data were acquired	Tweets and news on COVID-19 pandemic were retrieved using a set of keywords regarding to this topic. We used self-made Python scripts with both Twitter Streaming API and Requests API for Tweets and news, respectively.
Data format	Raw Analyzed Filtered
Parameters for data collection	Tweets and news matching a set of keywords in Portuguese, and from the start date of January until the end of May, 2020.
Description of data collection	We collected all data of Twitter and news articles posted from January to May, 2020, and filtered those in Portuguese, only. All the data are provided in csv-formatted text files. Data are provided together with sample Python code to read each dataset.
Data source location	News: Institution: UOL portal (www.uol.com.br) Country: Brazil Tweets: Institution: Twitter.com
Data accessibility	Repository name: Mendeley Data Data identification number: Published: 22 Jun 2020 Direct URL to data: https://data.mendeley.com/datasets/vhxdgjfjnk

**Value of the Data**

• These data are important because they are the first collection from two distinct popular sources from Brazil regarding the online discussion on COVID-19 pandemic.
• The dataset will be useful for researchers who want to conduct comparative studies on the perception of the pandemic from different media sources: formal news and social network posts.
• Academic institutions, public health agencies, scientific communities, researchers, students, and self-explorers can use these data and code to analyze the effects of COVID-19 in Brazil. Particularly, COVID-19 has severely affected Brazil, and it has generated high discussions from population in different points-of-view, such as health treatment, governmental recommendations, economical effects, mental-health and personal life issues. Thus, these data consists of material that can be applied in short or long term to assess people sentiment about pandemic, and for other important tasks to the broader public health community.
• These data were collected carefully from the beginning of the COVID-19 outbreak in January 2020. Thus, it is a timely dataset, which is considered as the additional value of our data.

## 1 Data Description

In this data article, we present a collection of 3,925,366 posts from Twitter and 18,413 online news gathered from UOL (https://www.uol.com.br) web site regarding the online discussion on COVID-19 in Brazil. These two media sources are the most popular sources for official and social information in Brazil. All the gathered data from Twitter were retrieved using a set of keywords in Portuguese: corona, coronavirus, COVID, COVID19, COVID-19, *distanciamento social* (social distancing), *isolamento* (isolation), lockdown, *quarentena* (quarantine), *ivermectina* (ivermectin), tamiflu, *cloroquina* (chloroquine), *azitromicina* (azithromycin), *hidroxicloroquina* (hydroxychloroquine), *pandemia* (pandemic), and *comorbidade* (comorbidity). These keywords were selected due to their popularity in web sites, such as Google Trends (https://trends.google.com.br), when associated to COVID-19. Also, all news were retrieved from COVID-19 sections of UOL web site. All the data were gathered from January 1 to May 31, 2020. To the best of our knowledge, it is the first public dataset of tweets and news on COVID-19 in Portuguese language and it has a considerable quantity of data.

**Fig. 1:**
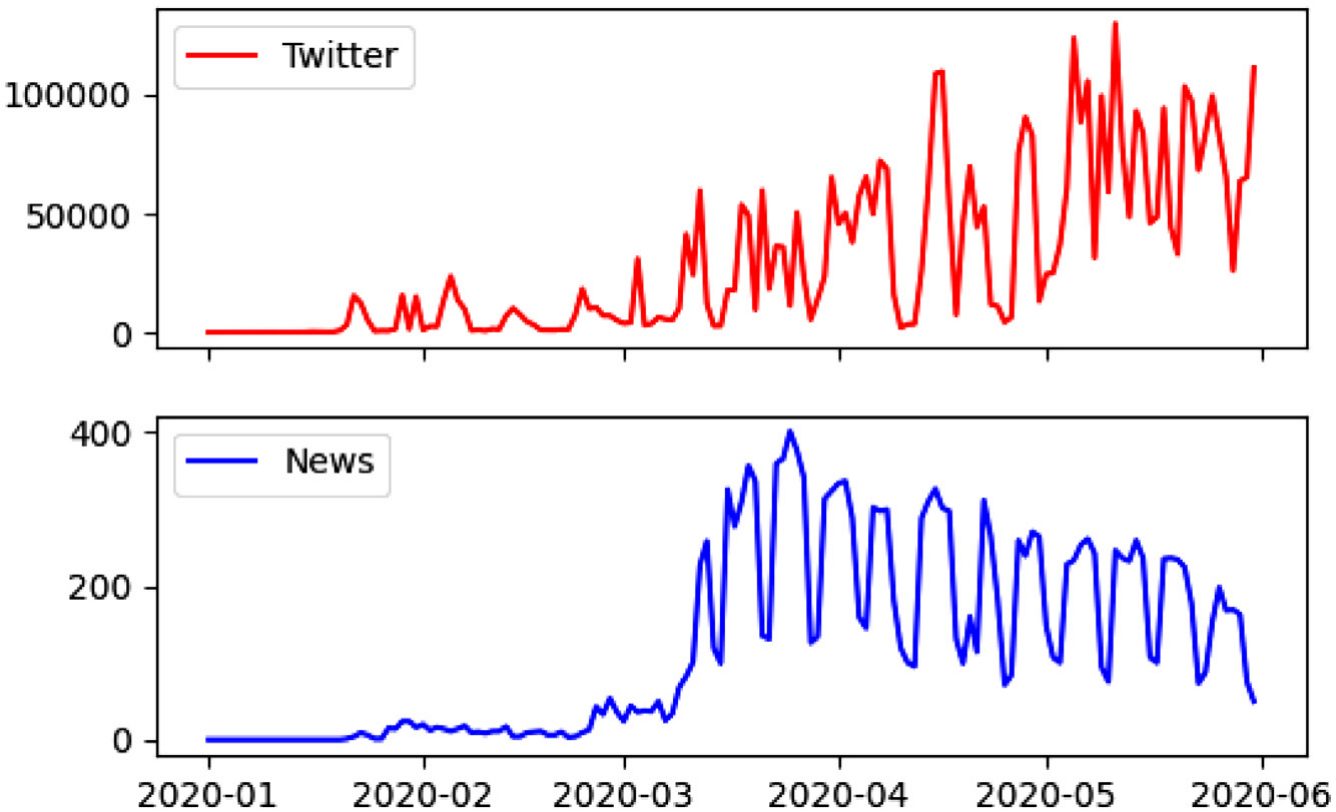
The trend of news and tweets regarding to COVID-19 topic.

Fig. 1 shows the daily distribution of the gathered tweets and news. We noticed that the discussions on COVID-19 topic across both platforms started to take off in late March. That occurred after the first death due to coronavirus in the Brazil [1]. Interestingly, the low points on the chart refer to weekends and holidays, showing a decrease on the number of posts by both users and journalists. Figure [1] also shows that the number of news published on COVID-19 in the last weeks of May is decreasing, while the number of tweets on this topic still remains high. The reason for this difference between the graphics may be due to the difference in interest between the two platforms on the same topic.

Since the raw data were huge, we had to filter and create different and specific datasets. All of them are available in a Mendeley dataset. Our data collection has been created according to Twitter's Terms & Conditions [2] and UOL's rules [3]. Table 1 presents the name of each dataset and a brief description of its fields. It is worth mentioning that all the datasets from Twitter contain the field *tweet_id,* which is a unique tweet identifier that can be used to join other data from Twitter from its API, like tweet metadata. The text news from UOL are available in its own dataset.

**Table 1:** Datasets in Mendeley.

Dataset	Description	Fields
General	Data collection of tweets regarding COVID-19. This dataset has approximately 220MB.	tweet_id: unique identifier for Twitter. keyword: term used to retrieve the tweets. date: when the tweet was created.
UOL	Data collection of news media regarding COVID-19. This dataset has approximately 65MB.	date: when the news media was posted in website. title: title of the news media URL: link to get the news media. text: actual text of each news media gathered.
Retweets	Data collection of tweet with at least one retweet. This dataset has approximately 26MB.	tweet_id: unique identifier for Twitter. screen_name: public username. number_retweets: number of retweets.
Media	Data collection of tweets with at least one picture or video. This dataset has approximately 31MB.	tweet_id: unique identifier for Twitter. media: link to visualize the media (picture or video)
Hashtags	List of hashtags in collected tweets. This dataset has approximately 32MB.	tweet_id: unique identifier for Twitter. tweet_hashtags: list of hashtags typed in tweet.
Python Scripts	List of programs written in Python to collect, transform, read and visualize each of the datasets. Each program has the following name format: 1) Collection - crawler-twitter.py and crawler-uol.py; 2) Transformation - create-<dataset_name>.py; 3) Reading - read-<dataset_name>.py; and 4) Visualizing – Script available at https://github.com/tmelo-uea/covid19	

## 2 Experimental Design, Materials and Methods

### 2.1 Data Source

In December 2019, the outbreak of COVID-19 in China was reported [4,5]. Due to the rapid spread of the virus in the world, the World Health Organization (WHO) declared a state of emergency. In Latin America, there were 937,974 cases of COVID-19 and 49,139 confirmed deaths until May 31, 2020 [6]. In South America, Brazil is the country most affected by the disease. According to the same report [6], there were 465,166 infected cases and 27,878 deaths in Brazil.

Due to the spread of the disease in the world, social media platforms and news web sites have become places where there is an intense and continuous exchange of information between government agencies, professionals and general public. A representative number of scientific studies have shown that social media and news sites can play an important role as a source of data for crisis analysis and also for understanding people's attitudes and their behavior during a pandemic [7,8,9].

In order to assist the monitoring of public health and also to support decision making by professionals, several monitoring systems have been developed to classify large amounts of data from social media. This data can be used to quickly identify the thoughts, attitudes, feelings and topics that occupy people's minds in relation to the COVID-19 pandemic [10]. Systematic analysis of these data can help policy makers and health professionals to identify issues of greatest interest to the population and resolve them in the most appropriate way.

### 2.2 Data Collection

**Fig. 2:**
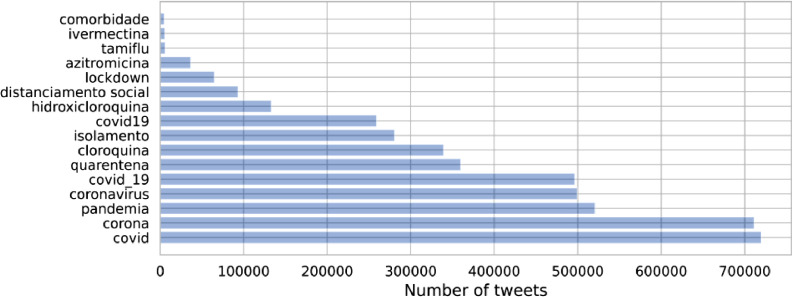
Distribution of tweets by keywords.

We collected news articles and tweets regarding COVID-19, in Portuguese, from January until May in 2020. For tweets collection, we used Twitterscraper (https://pypi.org/project/twitterscraper) Python library and we considered a set of keywords in Portuguese related to COVID-19 to filter the Twitter stream and obtain relevant tweets about the pandemic. The distribution of the collected data over for each keyword is shown in Fig. 2. In order to retrieve tweets only in Portuguese, we used the option *–lang* in Twitterscraper. Furthermore, we used the Google Translator to identify the language of collected tweets, and to discard those not in Portuguese. Twitter metadata presents a location entry, however, we noticed that very few users fill in this field and many of those users fill in non-standard labels. Thus, to keep a high number of tweets, we have chosen to filter by Portuguese language, once Brazil is the biggest country speaking this language (around 75% in the world^1^). Besides, we manually evaluated the tweets that informed the location, and only 4% (four percent) are from people who speak Portuguese and are not in Brazil.

**Fig. 3:**
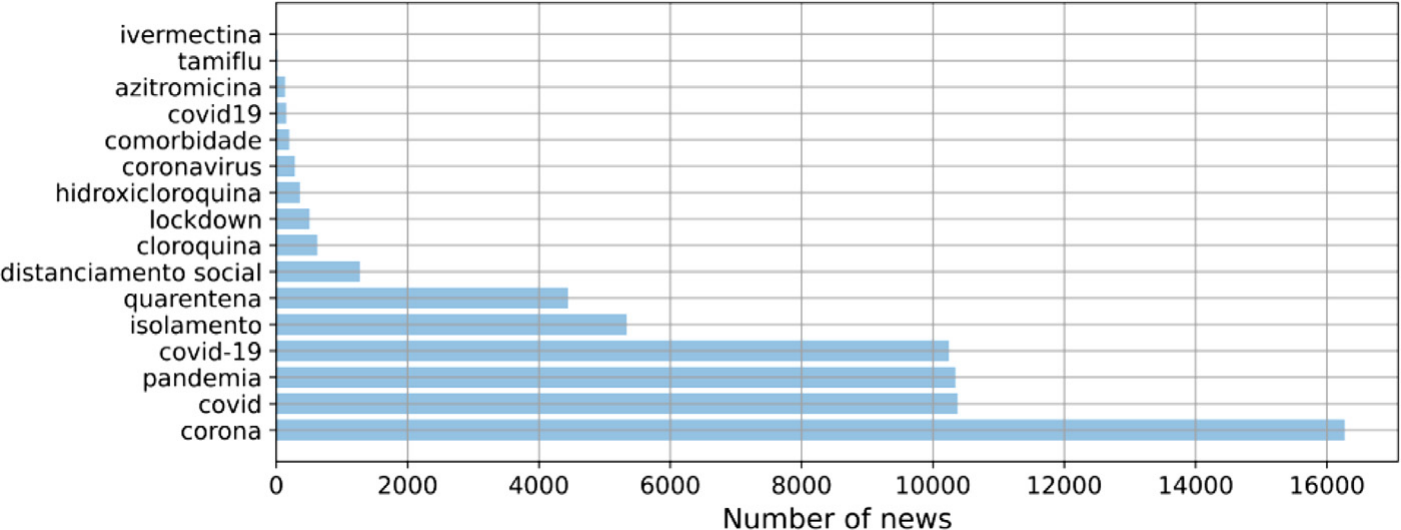
Distribution of news considering the same keywords for Twitter.

For news collection, we gathered all the articles published in a specific COVID-19 section from UOL portal, so we didn't have to filter by keywords. We have chosen UOL because it is responsible for publishing the *Folha de Sao Paulo* newspaper and it is the leading Brazilian newspaper by daily circulation [11]. Fig. 3 shows that the distribution of the news articles over each keyword is very close to the tweet collection. This indicates that there is a convergence of interest on a specific topic between journalists and social media users.

## 3 Hashtags

On Twitter, users post short public messages that are referred as tweets. These tweets can be sorted into categories by the inclusion of hashtags, or words or phrases beginning with a hash mark (#) and ending in whitespace, within the bodies of tweets. The use of hashtags (#) on Twitter allows followers to collate discussions around specific topics, including public health themes or events. In order to facilitate the identification of tweets that have hashtags for researchers or data enthusiasts, we created the dataset called Hashtags.

**Fig. 4:**
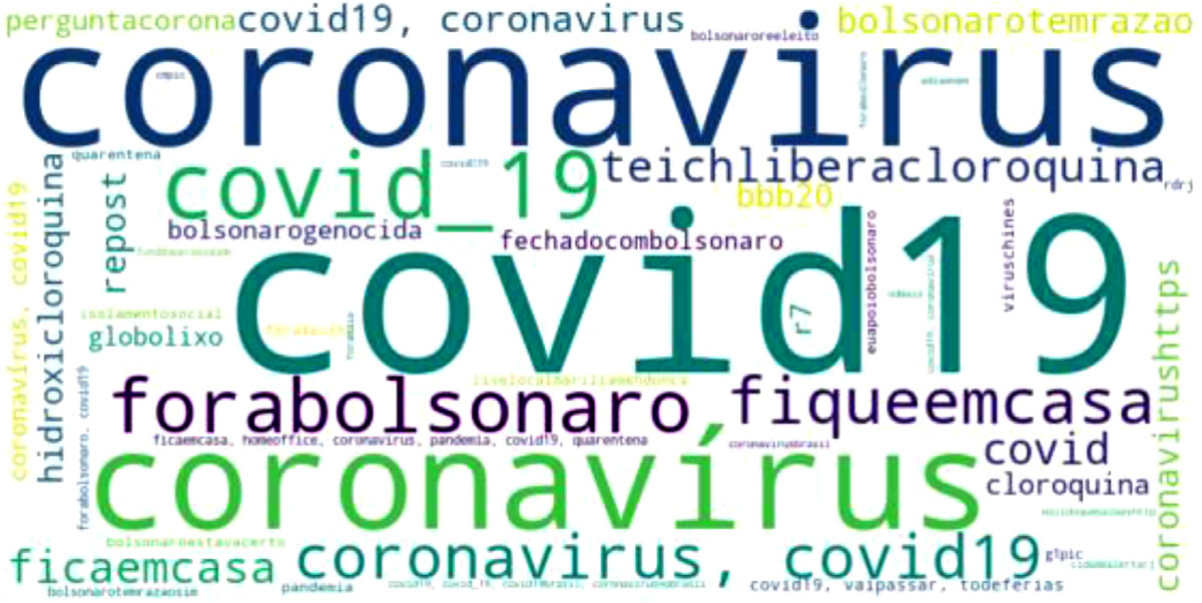
Wordcloud of hashtags used on Twitter on COVID-19 topic.

Wordcloud is a popular text analysis tool that provides a visualization of word frequency in the source text while giving more prominence to words that occur more often. Fig. 4 shows a wordcloud visualization of the 50 most frequently encountered hashtags in the dataset. It provides a general overview of the dominant terms related to the COVID-19 topic. We can observe that medical treatment, control procedures, and political issues are the most common themes discussed by users.

**Fig. 5:**
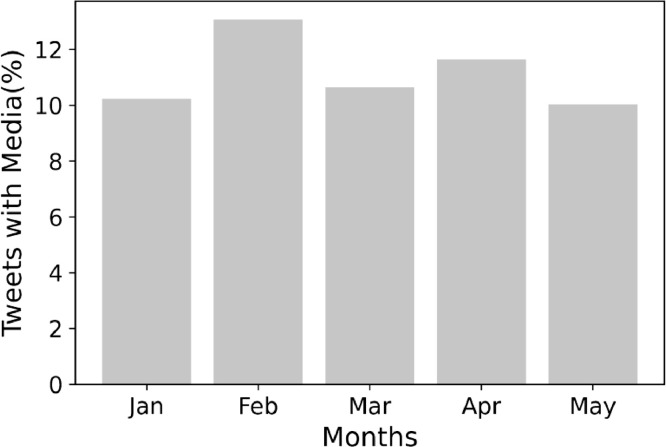
Percentage of tweets with image or video.

## 4 Multimedia Dataset

Twitter users usually post images and videos, in addition to texts. This type of content published in periods of crisis, such as pandemics and disasters, has been investigated by several researchers [12,13,14]. The image and video data produced during crises has potential value in helping researchers to understand the social experience during difficult times. In order to facilitate the identification of tweets that have images or videos for researchers or data enthusiasts, we created the dataset called Media. Figure [5] shows the percentage of tweets that were published with at least one image or video.

## 5 Corpus Analysis of the language

The impact of the current pandemic can be explored by looking at more frequent corpus terms in the Portuguese language. They are significantly more present in those months than in the corpus as a whole. Table 2 shows the 20 most frequent terms from January to May, 2020, and it was generated based on general.csv dataset. We can observe that they all are related to COVID-19, thus demonstrating that our queries were well chosen.

**Table 2:** Top 20 terms since January to May.

	January	February	March	April	May
1	coronavirus	coronavirus	COVID19	COVID19	pandemia
2	corona	quarentena	pandemia	coronavirus	COVID19
3	virus (virus)	corona	coronavirus	quarentena	social
4	China	vírus	vírus	pandemia	coronavirus
5	quarentena(quarantine)	China	social	social	isolamento
6	Brasil	COVID19	corona	isolamento	quarentena
7	pandemia (pandemic)	Brasil	quarentena	vírus	pessoas
8	casos (cases)	casos	isolamento (isolation)	corona	Brasil
9	pessoa (person)	pandemia	pessoas	Brasil	vírus
10	Saúde (health)	pessoas	cloroquina (chloroquine)	cloroquina	casa
11	mundo (world)	mundo	Brasil	COVID	meio (middle, kind or or media)
12	gente (people)	brasileiros (brazilians)	casa	saúde	saúde
13	novo (new)	carnaval	casos	pessoas	mortes (deaths)
14	Carnival (carnival)	Itália (Italy)	mundo	casos	mundo
15	medo (fear)	governo	gente	contra	contra
16	suspeita (suspicious)	país (country)	saúde	casa	distanciamento
17	cerveja (beer)	gente	Bolsonaro	Bolsonaro	COVID
18	surto (outbreak)	surto	COVID	mortes	cloroquina
19	governo (government)	casa (home)	lockdown	gente	Bolsonaro
20	cidade (city)	doença (disease)	contra (against)	mundo	president (president)

Although we used some drug names as keywords in queries, these terms were not as popular with users. This may indicate that people do not usually exchange information about medicines via Twitter.

## 6 Ethical Issue

In accordance with Twitter's Developer Policy [2], all data include only tweet IDs. User IDs and personally identifying information were removed from all tweet contents and metadata.

## Declaration of Competing Interest

The authors declare that they have no known competing financial interests or personal relationships which have, or could be perceived to have, influenced the work reported in this article.

## Appendix Supplementary materials

Image, application 1

## Footnotes

1 https://en.wikipedia.org/wiki/List_of_territorial_entities_where_Portuguese_is_an_official_language
